# Effect of HDI-Modified GO on the Thermoelectric Performance of Poly(3,4-ethylenedioxythiophene):Poly(Styrenesulfonate) Nanocomposite Films

**DOI:** 10.3390/polym13091503

**Published:** 2021-05-07

**Authors:** José A. Luceño-Sánchez, Ana Charas, Ana M. Díez-Pascual

**Affiliations:** 1 Universidad de Alcalá, Facultad de Ciencias, Departamento de Química Analítica, Química Física e Ingeniería Química, Ctra. Madrid-Barcelona Km. 33.6, 28805 Alcalá de Henares, Madrid, España (Spain); jose.luceno@uah.es; 2 Instituto de Telecomunicações, Instituto Superior Técnico, Av. Rovisco Pais, P-1049-001 Lisbon, Portugal; ana.charas@lx.it.pt

**Keywords:** graphene oxide, graphene-based polymer nanocomposites, hexamethylene diisocyanate, thermoelectrical properties, mechanical properties, organic solar cells

## Abstract

Composite films based on conducting polymers and carbon nanomaterials have attracted much attention for applications in various devices, such as chemical sensors, light-emitting diodes (LEDs), organic solar cells (OSCs), among others. Graphene oxide (GO) is an ideal filler for polymeric matrices due to its unique properties. However, GO needs to be functionalized to improve its solubility in common solvents and enable the processing by low-cost solution deposition methods. In this work, hexamethylene diisocyanate (HDI)-modified GO and its nanocomposites with poly(3,4-ethylenedioxythiophene):poly(styrenesulfonate) (PEDOT:PSS) were developed, and their morphology, thermal, electrical, thermoelectrical and mechanical performance were characterized. The influence of the HDI functionalization degree and concentration on the nanocomposite properties were assessed. The HDI-GO increased the crystallinity, lamella stacking and interchain coupling of PEDOT:PSS chains. A strong improvement in electrical conductivity, thermal stability, Young’s modulus and tensile strength was found, showing an optimum combination at 2 wt% loading. Drop and spin casting techniques were applied onto different substrates, and the results from deposition tests were analyzed by atomic force microscopy (AFM) and UV–vis spectroscopy. A number of parameters influencing the depositions process, namely solvent nature, sonication conditions and ozone plasma treatment, have been explored. This study paves the way for further research on conducting polymer/modified GO nanocomposites to optimize their composition and properties (i.e., transparency) for use in devices such as OSCs.

## 1. Introduction

Conducting polymers represent a class of organic polymers that can be semiconductors or exhibit metallic conductivity and typically possess advantageous properties for processing (e.g., light-weight, solubility or good dispersibility), which makes them very attractive for a wide range of applications in energy storage and conversion electronic [1,2,3,4,5,6]. The combination of conducting polymers with graphene-based materials has proved to be suitable for specific applications in some fields, in particular for organic solar cells (OSCs) and organic light-emitting diodes (OLEDs).

The exceptional optical, thermal and mechanical properties of graphene make it a potentially ideal filler to enhance the properties of polymeric matrices for diverse applications [7,8]. However, since graphene does not comprise any reactive functional groups, its surface is inert and its interaction with the polymer matrix is weak, this hindering its final processing. To circumvent such drawbacks, graphene can be modified via oxidation to yield graphene oxide (GO), which comprises oxygenated surface groups, namely epoxy and hydroxyl on the basal planes and carboxylic acids on the edges. GO is a water-soluble nanomaterial, and can be easily exfoliated in aqueous media upon sonication. However, the exfoliation of GO in organic solvents is restricted due to strong hydrogen bonding interactions between adjacent layers.

In this regard, a number of studies have focused on the chemical modification of GO to improve its dispersibility in organic media, which is crucial from a practical viewpoint, in particular for the development of polymer/graphene composites with easy processability and excellent performance for targeted applications [9,10,11,12]. If the extent of H-bonding in GO is minimized via functionalization, the layers become less hydrophilic, and more suitable for exfoliation in organic solvents. For instance, the addition of nucleophilic compounds, such as aromatic or aliphatic amines (e.g., pyrrolidine, ethylenediamine) yielded GO derivatives that could be homogeneously suspended in dimethyl sulfoxide (DMSO), N,N-dimethylformamide (DMF), water and ethanol, though they could not be dispersed in chloroform, benzene or toluene [13,14]. Polymers such as poly(ethylene glycol), poly(vinyl alcohol) and poly(methylmethacrylate) have also been anchored to GO via grafting-to or grafting-from approaches [15,16]. The former approach involves the anchoring of the polymer chain itself onto the GO surface, and can be performed via esterification, amidation, cycloaddition or click coupling reactions. Grafting-from methods are based on the anchoring of polymer-growth initiator molecules to the GO surface. Several grafting-from techniques have been described in the literature [17,18,19], the most important ones being Atom Transfer Radical Polymerization (ATRP), reversible addition fragmentation chain transfer polymerization (RAFT), polycondensation, ring opening polymerization and Ziegler–Natta polymerization. In general, the resulting functionalized GOs displayed better dispersibility in organic solvents, including dichlorobenzene [20], *N*-methyl-2-pyrrolidone (NMP) or DMSO.

Another attractive method is the isocyanate functionalization suggested in 2006 by Stankovich et al. [21]. The authors treated GO powder suspended in anhydrous DMF with several isocyanates, such as phenyl isocyanate, tert-butyl isocyanate and p-acetylphenyl isocyanate. The isocyanate treated GO was readily exfoliated in polar organic solvents such as DMF, NMP, and DMSO. However, it could not be dispersed in non-polar solvents, which limits its applications where non-polar solvents are required. In addition to isocyanates, other small organic molecules such as 1-ethyl-3-(3-(dimethyl amino)-1-propylamine)-carbodiimide [22], thionyl chloride [23] and *N*,*N*-Dicyclohexylcarbodiimide [24] have been used for GO functionalization. Octadecylamine (ODA) [25] has also been anchored to the carboxyl groups of GO, and the resulting alkylated GO showed good dispersion in organic solvents, such as tetrahydrofuran (THF) and carbon tetrachloride.

Currently, the most widespread conducting polymer is poly(3,4-ethylenedioxythiophene):poly(styrenesulfonate) (PEDOT:PSS), a polyelectrolyte containing negatively charged insulating PSS and positively charged electrically conducting conjugated PEDOT. PSS polymer anions can stabilize conjugated polymer cations in water and some polar organic solvents. This polyelectrolyte displays outstanding electrical conductivity, superior transparency (80–95%) in the visible range and very good flexibility, facile processing, cheap cost and low thermal conductivity, which make it a good candidate for hole transport layer covering the ITO electrode or even to replace the brittle and high-cost ITO electrode in OSCs [12]. However, as hole transport layer, PEDOT:PSS presents drawbacks such as the etching of the ITO electrode (due to being acidic) and its strong hygroscopic nature, which are detrimental to device efficiency and lifetime. Further, its thermoelectric performance is poorer than that of inorganic counterparts. To overcome these limitations, several researchers have studied composites made up of this conducting polymer and inorganic or carbon-based materials [26,27]. In the case of PEDOT:PSS/graphene nanocomposites, the π–π stacking interactions between the carbon-based nanomaterial and the polymer results in an improved electrical conductivity, chemical stability and thermoelectric performance [28,29,30]. These nanocomposites have been manufactured via solution mixing [26] or in situ polymerization of the EDOT monomer [27]. Nevertheless, it is difficult to homogeneously disperse graphene sheets in the polymer due to their low solubility in aqueous solution, which limits their practical applications. Therefore, novel efficient, inexpensive, simple and easy to scale up manufacturing techniques are still required.

In a previous study, chemically modified GO samples have been synthesized via treatment with hexamethylene diisocyanate (HDI) in the presence of triethylamine (TEA) as a catalyst [11]. The HDI-GO samples were able to form stable dispersions in a broad range of solvents with different polarities. In this work, PEDOT:PSS/HDI-GO nanocomposites with different HDI-GO functionalization degree and loading have been developed via solution casting method, and their morphology, thermal, electrical, thermoelectrical and mechanical behavior have been evaluated. Further, typical deposition techniques employed in the preparation of nanocomposites for organic thin film devices, namely drop casting and spin coating, have been tested, and the results were analyzed by UV–vis spectroscopy and atomic force microscopy (AFM) measurements. A number of parameters influencing the depositions process (i.e., solvent and substrate type, sonication conditions and ozone plasma treatment) have been examined.

## 2. Materials and Methods

### 2.1. Reagents

Commercially available PEDOT:PSS aqueous dispersion (Clevios PVP AI 4083, PEDOT:PSS concentration of 1.3–1.7 wt%, 1:6 PEDOT:PSS weight ratio, η = 12 mPas, average particle size = 80 nm), was purchased from Heraeus Electronic Materials (Germany). H_2_SO_4_, KMnO_4_, P_2_O_5_, K_2_S_2_O_8_ and H_2_O_2_ were acquired from Sigma-Aldrich. Graphite powder was obtained from Bay Carbon, Inc. For the synthesis of HDI-GO, triethylamine (TEA, >98%, M_W_ = 101.193 g/mol) and hexamethylene diisocyanate (HDI, >99%, M_W_ = 168.196 g/mol) were purchased from Acros Organics. HPLC grade organic solvents were acquired from Scharlau S.L. (Barcelona, Spain). The deionized water was produced with a Milli-Q-Water-Purification-System. All the chemicals were employed without further purification.

### 2.2. Synthesis of GO and HDI-GO

The preparation of GO was carried out using a modified Hummers’ method from graphite powder as reported elsewhere [31]: 2 g of graphite powder was heated with 15 mL of H_2_SO_4_, 2.5 g of P_2_O_5_ and 2.5 g of K_2_S_2_O_8_, and then deionized water was added. The product was filtered and mixed with 15 g of KMnO_4_, 30 mL of H_2_O_2_ and 120 mL of H_2_SO_4_. The solution was heated to 80 °C in an oil-bath and stirred for 24 h. The final product was purified by centrifugation, followed by several cycles of purification with H_2_O_2_/H_2_SO_4_ washing, bath ultrasonication, and finally washed with deionized water followed by vacuum-drying.

**Scheme 1:**
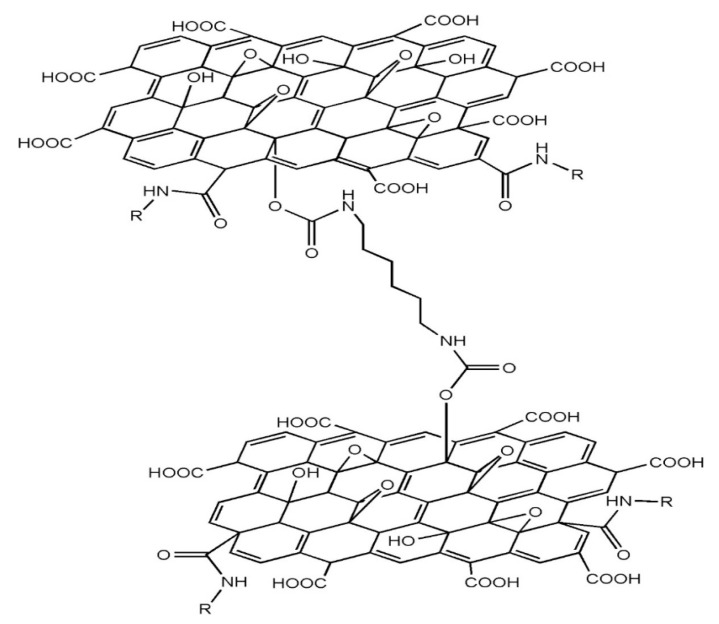
Hexamethylene diisocyanate (HDI)-functionalized GO structure.

The synthesis of HDI-GO was achieved by applying the procedure described in previous works [11], which can be summarized as follows: (1) GO powder (ca. 250 mg) was probe sonicated and subsequently ultrasonicated in an ultrasonic bath using toluene as solvent. (2) TEA (ca. 8.75 mL) and HDI (5 mL) were added dropwise to the GO dispersion and the mixture was heated and stirred overnight under inert atmosphere. (3) The resulting mixture was coagulated with methylene chloride, filtered, washed repeatedly and dried under vacuum. The chemical structure of the synthesized HDI-GO is shown in Scheme 1. The reaction conditions, namely reaction time and temperature, GO/HDI/TEA weight ratio, tip/bath sonication cycles and solvent volume, were modified, and samples with FD between 3.1 and 18.1 moles of carbamate ester unit incorporated per mol of carbon atoms of GO were attained. To study the effect of FD on the dispersion properties, two HDI-GO samples were chosen: (a) one with FD of 3.12%, labeled as HDI-GO 4 (reaction time of 12 h, reaction temperature of 90 °C, 1:1:1 GO/HDI/TEA weight ratio, 25 mL of solvent and 120 min of bath sonication); (b) the other with FD of 17.20%, named as HDI-GO 5, synthesized under the same conditions except for 60 °C reaction temperature, 50 mL of solvent and a previous ultrasonic tip treatment of 5 min.

### 2.3. Preparation of PEDOT:PSS/HDI-GO Nanocomposites

**Figure 1:**
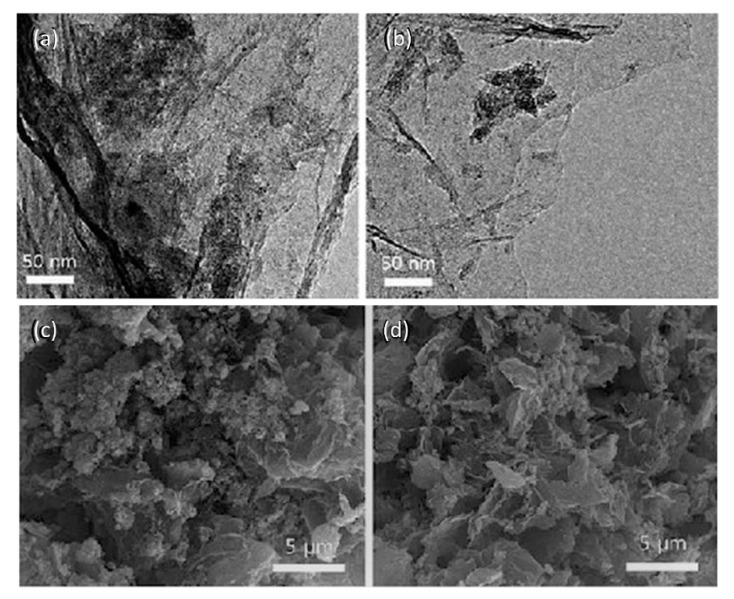
TEM (**top**) and SEM (**bottom**) images of PEDOT:PPS/HDI-GO5 nanocomposites with HDI-GO loadings of 10 wt% (**a**,**c**) and 1.0 wt% (**b,d**).

Nanocomposites with HDI-GO loadings of 0.5, 1.0, 2.0, 5.0 and 10 wt% were prepared by means of solution casting technique. In short, the required amount of HDI-GO was suspended in DMSO by ultrasonication in a bath for 1 h. Subsequently, the PEDOT:PSS aqueous dispersion was slowly added to the HDI-GO suspension, and the mixture was ultrasonicated for additional 2 h, casted onto Petri dishes and dried in an oven. Partly heterogeneous thin films with an average thickness of 300 nm were attained [11], as revealed by microscopic images (SEM and TEM, Figure 1). The HDI-GO was not completely suspended in DMSO by bath sonication; hence, some small aggregates could be observed. However, the polymeric chains were able to wet the stacking structure of modified GO, leading to the formation of a thin polymeric coating (Figure 1a,b). The HDI functionalization makes GO surface more hydrophobic, hence suitable to interact with neutral PEDOT segments and the alkyl side chains of PSS via van der Waals forces. Further, the positively charged PEDOT segments can interact with HDI-GO via electrostatic interactions with deprotonated carboxylic groups as well as via π–π stacking between their double bonds and the aromatic graphene rings. In addition, negatively charged sulfonyl groups of PSS are prone to interact with hydroxyl groups of HDI-GO via H-bonding, and all these strong interactions result in a nanofiller fully embedded within the matrix.

### 2.4. Layer Deposition

Firstly, HDI-GO samples were suspended in different solvents (NMP, 2-propanol (IPA) and DMSO) at a concentration of 10 wt% via sonication for 1 h. Then, they were deposited by two different methods: drop casting and spin coating. The samples were deposited onto glass substrates, except some spin coating tests that employed glass/ITO substrates. In the case of PEDOT:PSS/HDI-GO nanocomposites (HDI-GO loading of 10 wt%) layer deposition, the preparation is analogous to HDI-GO ones: the mixture is sonicated prior to deposition. To investigate the effect of the ultrasonication conditions on the film transmittance, two sets of samples were prepared for each HDI-GO: (1) control set, in which the ultrasonication conditions are identical to those of previous works (2 h) [12]; (2) double cycle set, which involved a first stage of 1 h, a rest of 12 h followed by a second stage of 2 h.

Drop casting conditions were similar to those described above for the preparation of PEDOT:PSS/HDI-GO nanocomposites. For this approach, two sets of experiments were carried out: (1) HDI-GO 4 and HDI-GO 5 samples, at a concentration of 2 mg/mL); and (2) PEDOT:PSS/HDI-GO 5 samples with a HDI-GO loading of 10 wt%. Each set of experiments was also tested using two solvents: DMSO and NMP, which were chosen based on the results of previous studies [11,12]. A thermal annealing step was applied to dry the samples.

Spin coating was carried out at varying speeds to tentatively obtain layers with different thicknesses. In this case, dispersions of HDI-GO 5 in DMSO (10 wt%) were chosen, since this functionalized nanomaterial showed the best dispersibility in this solvent [11]. Two different spin coating speeds were chosen, 1000 rpm and 1800 rpm; the coating deposition time (15 s) was kept constant for all the samples, and the drying speed (1000 rpm) and drying time (45 s) also. The experimental conditions used for the spin-coating are detailed in Table 1.

For all the spin-coated samples, the surface was treated with an ozone plasma for 6 min, approximately, in order to reduce its hydrophobicity and improve the deposition of aqueous dispersions, such as PEDOT:PSS. This treatment was also used to clean the surface from any contaminants. The samples were introduced into a chamber using a sample holder, and then sealed; subsequently, a vacuum was made inside, and a flow of oxygen was introduced while UV light was shed into the chamber to generate the ozone plasma.

### 2.5. Instrumentation

Samples were weighed in a Mettler Toledo AB204 Balance with readability of 0.1 mg. Ultrasonication was performed with a P Selecta Ultrasons 59606 bath, at 50/60 Hz and a maximum power output of 50 W. Substrates (bare glass 12 mm × 12 mm × 1 mm; ITO/glass 12 mm × 12 mm × 1 mm) were washed with non-ionic detergent and deionized water in an ultrasonic bath, dried and cleaned with a stream of N_2_.

Scanning electron microscopy (SEM) images were acquired with an SU8000 Hitachi scanning electron microscope, at a voltage of 15.0 kV and emission current of 10 mA. Prior to the observations, the films were cryo-fractured in liquid nitrogen and then coated with a ∼5 nm Au:Pd overlayer to avoid charge accumulation during electron irradiation. Transmission electron microscopy (TEM) images were acquired using a Zeiss EM-10C/CR instrument at a voltage of 60 kV, with a magnification of 500,000×.

Ozone plasma was generated with O_2_ Plasma cleaner GaLa Instruments Plasma Prep2 2004 equipped with a vacuum pump.

Silver conductive paint was used to create top and bottom contacts for the Seebeck coefficient and electrical conductivity measurements, which were carried out on a ZEM-3M8 ULVAC System (Advanced Riko, Inc., Yokohama, Japan) under RT and a helium environment. Five specimens for each composition were tested in order to report an average value.

Tensile tests under RT conditions were performed with an Instron 5565 Testing Machine (Norwood, MA, USA), using a 1 kN load cell and at a crosshead speed of 10 mm/min. The results reported are the mean values for six replicates.

The thermal stability of the samples was investigated via thermogravimetric analysis (TGA) with a TA Instruments Q50 thermobalance (Barcelona, Spain), at a heating rate of 10 °C/min, from room temperature to 700 °C. After drying for 72 h, about ~5 mg of each sample were placed into an alumina pan and measured under an inert atmosphere, with a purge gas flow rate of 60 mL/min.

A Bruker D8 Advance diffractometer (Karlsruhe, Germany) was used to perform the X-ray diffraction (XRD) analysis. A Cu tube was employed as the X-ray source (Cu-Kα = 1.54 Å), with a voltage of 40 kV and an intensity of 40 mA.

UV–vis spectra were recorded using a Cecil 7200 spectrophotometer in the 250 to 900 nm wavelength range, at room temperature. The thermal annealing of drop-casted and spin-coated layers was carried out with a conventional heating plate, under air. The spin coating was performed using a Spin coater Chemat Technology Kw-4A 2000 connected to a vacuum pump.

Atomic Force Microscopy (AFM) was carried out with a Nano-Observer-Model 5100 microscope equipped with a digital AFM Nano-Observer CSInstruments controller. As HDI-GO and the polymers can be considered as soft materials, images were acquired using the non-contact mode. ACT-50 App Nano silicon tips with ca. 10 nm tip ratios were used as probes.

## 3. Results and Discussion

### 3.1. XRD Patterns of HDI-GO/PEDOT:PSS Nanocomposites

**Figure 2:**
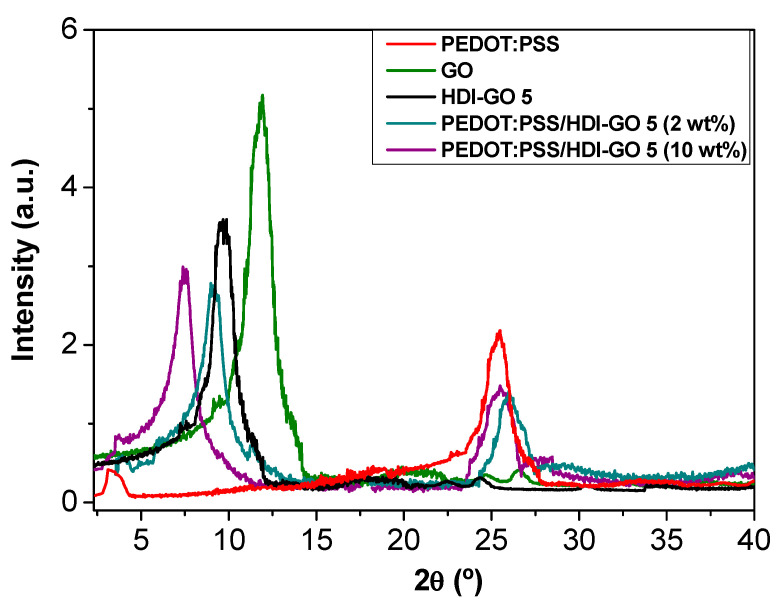
XRD patterns of neat PEDOT:PSS, reference GO, HDI-GO 5 and PEDOT:PSS/HDI-GO 5 nanocomposites with 2 and 5 wt% loading.

To get insight about the effect of HDI-GO on the structure and ordering of the polymeric chains of PEDOT:PSS, samples were analyzed via XRD measurements, and typical patterns of PEDOT:PSS/HDI-GO 5 and the corresponding nanocomposites with 2 and 5 wt% loading are compared in Figure 2. Similar diffractograms were obtained for the rest of HDI-reinforced nanocomposites, and the data derived from their diffraction patters are summarized in Table 2.

For raw PEDOT:PSS, which shows semicrystalline nature, two main peaks can be observed at 2θ values of approximately 3.5° and 25.5° which correspond to lattice d spacings of 25.21 Å and 3.49 Å, respectively, calculated according to the Bragg’s law [32]: λ = 2d sin θ. The d spacing of 25.21 Å has been ascribed to the distance between the lamella stacking *d*(100) due to the alternate ordering distance of PEDOT and PSS [33]. This lamella stacking distance is in good agreement with the width of the PEDOT chain (7.5 Å) obtained via structural simulation [34]. On the other hand, the reflection at 2θ = 25.5° is attributed to the inter-chain planar ring-stacking distance of *d*(010) of PEDOT.

Regarding the nanocomposites, a reduction in the lamella stacking distance is observed (i.e., from 25.21 to 22.06 Å for the sample with 2 wt% HDI-GO5), and simultaneously the π–π stacking distance somewhat decreases from 3.49 to 3.41 Å (Table 2). This diminution in the π–π stacking distance could be related to a transformation of the PEDOT and PSS chains from benzoid to quinoid structure and therefore become more planar upon incorporation of HDI-GO [35]. In addition, the distance between rings of different PEDOT chains decreases, indicating a denser and more closely packed structure. Interestingly, for composites reinforced with either HDI-GO 5 or HDI-GO 4, the lamellar stacking and inter-chain planar ring-stacking distances decrease to a minimum value at a concentration of 2 wt% loading and then rise slightly, hinting that this nanomaterial concentration is optimal to attain a closely packaged structure. Further, the reduction is slightly more prominent upon addition of the HDI-GO with higher FD.

On the other hand, raw GO shows a characteristic peak at 2θ = 11.8 related to the (002) diffraction of the GO sheet [36], corresponding to a *d* spacing of 0.748 nm. For HDI-GO 4 and HDI-GO 5, the reflection has lower intensity and appears at 2θ = 10.7° and 9.9°, indicating higher interlayer distance. The growth in the d spacing is likely induced by the HDI chains intercalated between the GO layers, and has been previously observed for nanocomposites with polymeric chains inserted between the GO nanosheets [37]. Overall, the nanocomposites exhibit sharper peaks in the low angle region, implying a higher crystallization degree of the PEDOT:PSS. All the XRD results suggest that the incorporation of HDI-GO increases the level of crystallinity, the lamella stacking between two assemblies and the interchain coupling of PEDOT:PSS chains, with a more densely packed PEDOT [34].

### 3.2. Thermoelectric Performance of HDI-GO/PEDOT:PSS Nanocomposites

**Figure 3:**
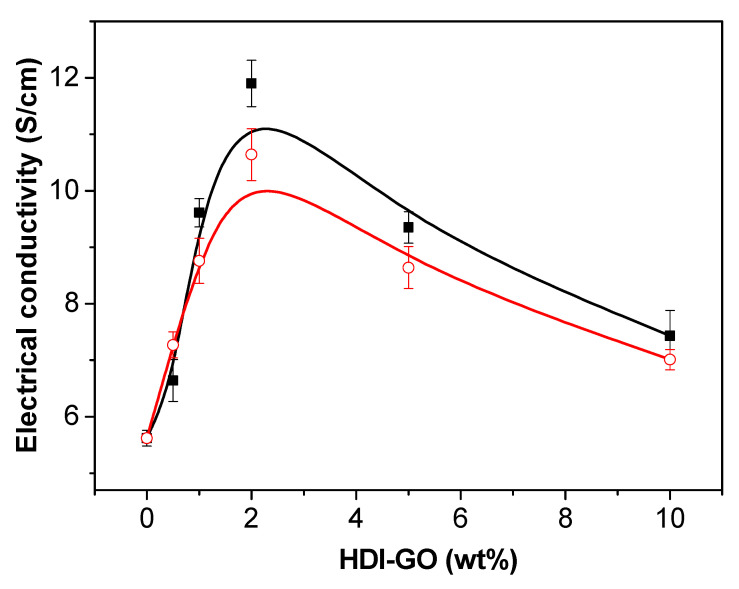
Electrical conductivity of PEDOT:PSS nanocomposites reinforced with HDI-GO 5 (red open circles) and HDI-GO 4 (black solid squares) as a function of the HDI-GO loading.

Figure 3 shows the effect of HDI-GO concentration and functionalization degree on the electrical conductivity of PEDOT:PSS. Noticeably, the addition of small amounts of the modified carbon nanomaterial brings a strong improvement in the electrical conductivity of the polymer matrix, despite the lower electrical conductivity of GO [38]. Thus, more than 2-fold increment is attained for an HDI-GO4 loading of 2.0 wt%. This improvement should be ascribed to the strong interactions between the graphene derivative and the polymer via π–π stacking, H-bonding and hydrophilic–hydrophobic, in agreement with results reported earlier for PEDOT:PSS nanocomposites reinforced with graphene quantum dots (GQDs) [39]. Given that the size of PEDOT:PSS chains is larger than that of the modified carbon nanomaterial, the conductive network depends on the close connection among the polymeric segments, therefore improving the electrical conductivity of the whole matrix. Thus, the presence of the HDI-GO can influence the charge hopping conduction mechanism in this conductive polymer via doping and screening effects as well as inducing conformational changes of the polymeric chains [40], as inferred from XRD analysis. The positively charged PEDOT segments and negatively charged PSS segments are assembled via Coulomb attractions, and show a coiled or core–shell structure due to repulsion among PSS chains. These electrostatic forces may be screened via formation of hydrogen bonds between the surface OH groups of HDI-GO and the sulfonyl groups of PSS, leading to a more densely packaged PEDOT chains on the nanocomposite surface [41], as confirmed by the decrease in the inter-chain planar ring-stacking distance of this polymer derived from XRD patterns (Table 2). Thus, the minimum *d*(100) distance is obtained for the nanocomposites with 2.0 wt% HDI-GO (for both FD), which show the maximum conductivity (Figure 3). Further, taking into account that the matrix is a semicrystalline polymer, the increase in the level of crystallinity upon addition of the HDI-GO would result in higher conductivity. Thus, it has been reported [42] that the addition of conductive nanofillers increases the conductivity since they concentrate in the amorphous (inter-crystalline) region, thus favoring the carrier hopping and transport between PEDOT chains.

However, for both FD tested, the conductivity decreases at HDI-GO loadings higher than 2–5 wt%, attributed to the partial aggregation of the carbon nanomaterial in the polymer matrix, as revealed by SEM and TEM images. This is consistent with the results reported previously for PEDOT:PSS reinforced with graphene [43] or its derivatives [39]. Further, the addition of high concentrations of a nanomaterial with lower conductivity than the matrix could have a detrimental effect on the electrical conductivity of the whole sample. Noticeably, for the same nanofiller content, the conductivity is systematically higher for nanocomposites comprising HDI-GO 4, the derivative with lower FD, despite that it is less uniformly dispersed within the matrix. This can be rationalized considering that this derivative has more residual surface OH groups capable of interactions with the sulfonyl groups of PSS, hence the abovementioned screening effect will be stronger. Further, the electrical conductivity of HDI-GO 4 should be higher than that of HDI-GO 5, since the HDI functionalization treatment partly disrupts the aromatic π-system of the GO nanosheets. The electrical conductivity values obtained herein are comparable to those previous reported for PEDOT:PSS nanocomposites with GO or GQDs [38], corroborating that the approach using herein is also beneficial for improving the matrix conductivity.

**Figure 4:**
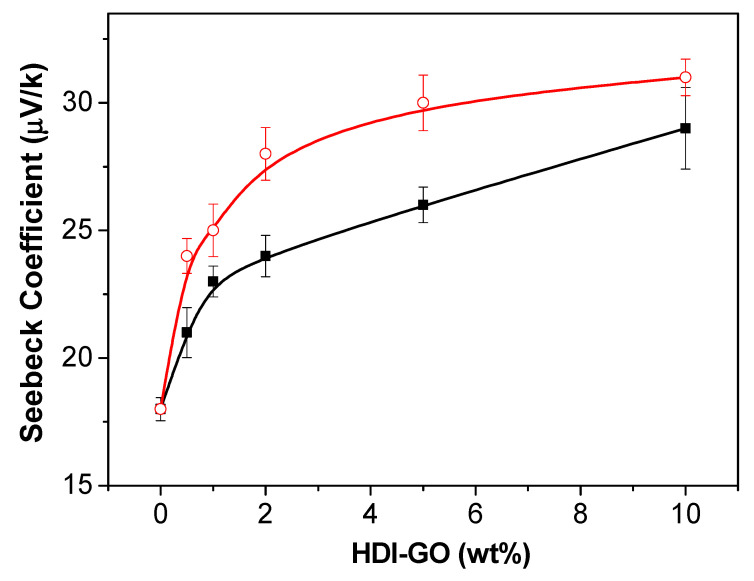
Seebeck coefficient of PEDOT:PSS/HDI-GO nanocomposites as a function of the HDI-GO loading. Symbols as indicated in Figure 3.

The Seebeck coefficient of a material, sometimes referred to as thermopower or thermoelectric power, is a measure of the magnitude of an induced thermoelectric voltage in response to a temperature gradient across that material, which is induced by the Seebeck effect (one of the thermoelectric effects) [44]. It measures the efficiency of a material to convert heat directly into electrical energy. The experimental values of the Seebeck coefficient for the PEDOT:PSS nanocomposites reinforced with HDI-GO 4 and HDI-GO 5 are compared in Figure 4. As can be observed, all the nanocomposites have Seebeck coefficients higher than raw PEDOT:PSS. This parameter steadily increases with increasing nanofiller loading, the rise being more prominent at low loadings.

Zhang et al. [45] have described that nanostructured fillers in a PEDOT:PSS matrix can improve the Seebeck coefficient by the filtration of low-energy carriers and transporting high-energy carriers, named as “energy-filtering effect”. The same mechanism could apply to the HDI-GO reinforced nanocomposites. Thus, the strong interactions between the carbon nanomaterial and PEDOT segments via π–π stacking could result in polymeric segments orderly aligned on the HDI-GO surface, which improves the electrical conductivity. On the other hand, the assembly of hydrophilic groups between HDI-GO and PSS could favor the quick transport of carriers. In addition, the formation of a more densely packed and a higher degree of crystalline structure as evidenced by the sharper XRD diffractions at low diffraction angles as well as a shorter π–π stacking distance would be reflected in increased Seebeck coefficient [33]. In addition, the strong interactions at the molecular level at the HDI-GO/PEDOT:PSS interphase could lead to an energy filtering effect, further improving the Seebeck coefficient.

This would result in improved thermoelectric properties for the nanocomposites, in particular those with higher FD, which are better dispersed within the matrix, hence the interactions at the molecular level should be stronger. This is consistent with previous studies, which found that graphene and its derivatives not only improve the electrical conductivity of conductive polymers, but also increase the Seebeck coefficient owed to the energy filtering and ordered chains at the interphases within the nanocomposites [46,47]. It is important to note that the Seebeck coefficient obtained herein for the nanocomposite with 5 wt% HDI-GO 5 is larger than that obtained upon addition of the same amount of pristine graphene. This can be explained considering that raw graphene, as a zero band-gap material, has a small Seebeck coefficient [48]. However, it has been shown both theoretically and experimentally that by introducing an array of holes into the graphene sheet a band-gap can be achieved [49]. Thus, the HDI functionalization treatment introduces defects in the graphene network, and this in turn would modify the band structure of the nanomaterial, and hence increase the Seebeck coefficient.

### 3.3. Thermal Stability of HDI-GO/PEDOT:PSS Nanocomposites

**Figure 5:**
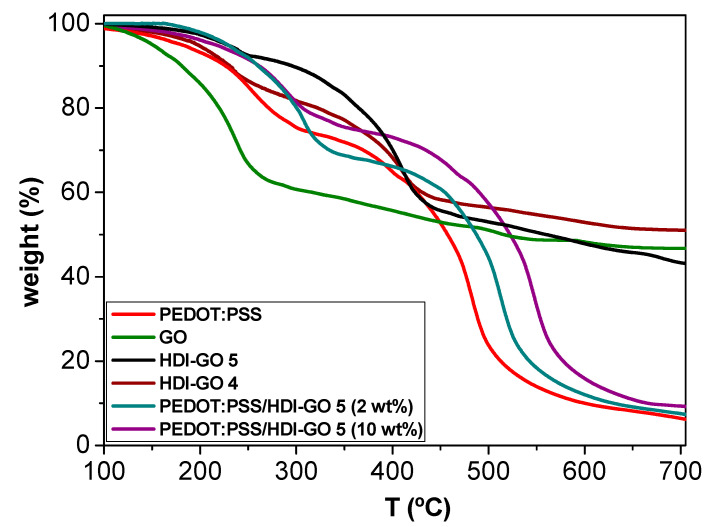
TGA curves under inert atmosphere of neat PEDOT:PSS, reference GO, HDI-GO 4, HDI-GO 5 and the nanocomposites with 2 and 10 wt% loading.

Thermal stability of polymer composites is of great interest for certain applications such as thermoelectric devices. To obtain information about the thermal stability of the PEDOT:PSS/HDI-GO nanocomposites, TGA measurements were performed under an inert atmosphere, and the results for nanocomposites with 2 and 5 wt% loading are shown in Figure 5. Data derived from the rest of the nanocomposites are collected in Table 2. Pristine PEDOT:PSS shows two decomposition stages; the first weight loss up to 300 °C can be accredited to the decomposition of PSS through elimination of the sulfonate groups [50], and the second weight loss up to 550 °C is ascribed to the disruption of the PEDOT and/or PSS backbone chain [51]. On the other hand, raw GO displays a single-step degradation process, with a mayor weight loss below 250 °C due to the decomposition of the surface epoxide, hydroxyl and carboxylic acid groups. Furthermore, a small weight loss is found above 260 °C owed to the elimination of additional functional groups.

Regarding the HDI-GOs, two decomposition stages can be observed, one up to about 440 °C due to the removal of the residual oxygenated groups on the GO surface, and the second to the decomposition of the HDI chains linked to the GO. Interestingly, HDI-GO 5 with higher FD shows slightly better thermal stability, likely due to the higher degree of crosslinking between the nanosheets, since the increase in the cross-linking degree typically results in improved heat resistance [52].

Focusing on the PEDOT:PSS/HDI-GO nanocomposites, a two-step degradation process is also observed, similar to that of pristine PEDOT:PSS. With the addition of increasing HDI-GO contents, the TGA curve shifts progressively to higher temperatures, and both the initial degradation temperature (T_i_) and the temperature of the maximum rate of weight loss (T_max_) rise, as well as the weight residue (Table 2), revealing higher thermal stability and flame resistance. For both temperatures, the maximum improvement (74 and 51 °C, respectively) is found at 5 wt% loading, likely because more aggregates are present at higher concentrations that decrease the barrier effect of the functionalized GO. Thus, the crosslinked HDI-GO sheets homogeneously dispersed within the conductive polymer (Figure 1) can behave as a barrier and delay the flow of the degradation products from the bulk of the sample to the gas phase via formation of a tortuous pathway. Further, it could also act as a thermal shielding material to insulate the PEDOT:PSS chains from the heat. Similar behavior has been reported for other polymeric composites reinforced with functionalized GO [38,53], ascribed to the free radical transfer between the matrix and graphene nanosheets and the barrier effect of functionalized GO. Further, the strong PEDOT:PSS-HDI-GO interactions could restrain the rotational movement of the polymeric chains, thus decreasing molecular movement, which is reflected in better thermal stability.

It can be inferred from Table 2 that the thermal stability increases with increasing the FD of HDI-GO, attributed to the higher crosslinking between the GO layers and the stronger interactions between chains, that results in a more effective barrier effect. However, composites of 10 wt% HDI-GO show slightly lower stability than those with 5 wt% concentration, suggesting that the barrier effect imposed by the nanomaterial layers has leveled off. Overall, it is found that the addition of HDI-GO significantly improves the thermal stability of PEDOT:PSS, which is crucial from a practical viewpoint.

### 3.4. Tensile Properties of HDI-GO/PEDOT:PSS Nanocomposites

**Figure 6:**
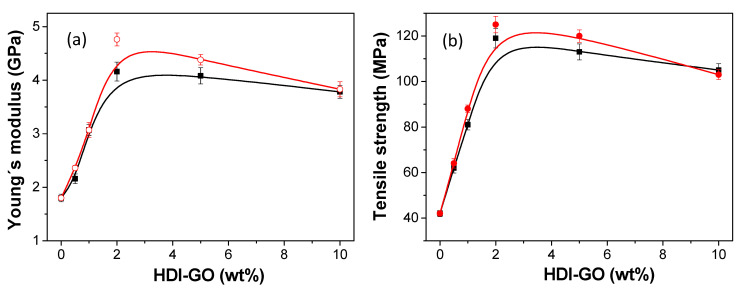
Young’s modulus (**a**) and tensile strength (**b**) of PEDOT:PSS/HDI-GO as a function of the HDI-GO loading. Symbols as indicated in Figure 3.

Tensile tests were performed to get insight into the reinforcement effect induced in the conductive matrix upon addition of HDI-GO, and the Young’s modulus and tensile strength as a function of HDI-GO loading for the two FDs tested are compared in Figure 6. Neat PEDOT:PSS exhibits a Young’s modulus close to 1.8 GPa, in agreement with the results found by other authors [54]. The addition of HDI-GO 5 causes a noticeable stiffness increment, by more than two-fold at 2 wt% loading, albeit the reinforcement effect decreases slightly at higher loadings probably due to the presence of small aggregates. The strong modulus increase found at low loadings demonstrates the great reinforcing efficiency of HDI-GO, especially that with the highest FD, likely due to the combination of a random and uniform nanomaterial dispersion within the matrix and a very strong PEDOT:PSS-HDI-GO interfacial adhesion reached by hydrogen bonding, electrostatic, hydrophobic and π–π interactions, as mentioned above. In fact, it has been reported that the mechanical properties of graphene-polymer nanocomposites are controlled by interactions on the molecular scale between the nanomaterial and the polymer matrix [55]. Further, other effects such as crystal nucleation and molecular confinement can also be crucial in enhancing the nanocomposite stiffness. Stress transfer from the matrix to the exfoliated nanosheets is assumed to take place through a shear stress at the nanosheet/matrix interface [56]. Since the GO nanosheets are reported to have much higher Young’s modulus than the matrix, they would carry most of the load in the nanocomposites.

In the case of nanocomposites containing graphene nanosheets, the Young´s modulus can be predicted by the Krenchel’s rule of mixtures [57]: E_c_ = (η_o_η_l_E_f_ − E_m_)V_f_ + E_m_, where η_o_ is the orientation factor, η_l_ the length factor, E_f_ the filler modulus, E_m_ the matrix modulus and V_f_ the filler volume fraction. η_o_ = 8/15 for randomly oriented nanosheets; the length factor (0 ≤ η_l_ ≤ 1) reflects the efficiency of the stress transfer from the matrix to the filler that is controlled by both the shape of the filler and the strength of the filler–matrix interface. Taking reported data for the Young’s modulus of GO (~207 GPa [58]), it is possible to estimate the theoretical values for the concentration range studied. Thus, experimental data of composites with loadings < 2 wt% are in very good agreement with the predictions (deviations lower than 8%). However, the nanocomposite with 2 wt% HDI-GO 5 shows about 17% higher modulus than the theoretical predictions, suggesting that other factors not taken into account by the equation like an increase in the matrix crystallinity due to nucleation effects could also influence the composite modulus. On the contrary, the values of nanocomposites with loadings > 2 wt% are lower than the predictions, likely due to the presence of aggregates that reduce the PEDOT:PSS-HDI-GO interfacial area and limit the load transfer efficiency. In addition, the crumpling of graphene nanosheets and the presence of different types of defects can decrease significantly the stiffness. Thus, new theoretical models should be developed in order to better describe the experimental results. In the theoretic perspective, fractal theory is a very important tool, which can be used to investigate the morphology and mechanical performance of polymer/nanocomposite films [59,60].

Noticeably, the reinforcing effect reached upon addition of HDI-GO 5 is comparable to that observed for PEDOT:PSS nanocomposites filled with polyethylene glycol (PEG)-modified single-walled CNTs, despite the higher modulus of the CNTs (about 1 TPa) [61] compared to GO. All these facts corroborate the effectiveness of the HDI treatment to enhance the mechanical performance of PEDOT:PSS.

Regarding the tensile strength, the trend observed is very similar to that of the modulus, with a very strong increase (up to 3-fold) at low concentrations and a level off or even decrease at higher loadings. This raise in the tensile strength is also ascribed to the strong interfacial adhesion due to the numerous polymer-nanofiller interactions (H-bonding, π–π stacking, electrostatic and so forth), as mentioned above.

### 3.5. Deposition of HDI-GO and HDI-GO/PEDOT:PSS Dispersions

With a view to use the developed nanocomposites in thin film devices using solution deposition methods, two deposition techniques, drop casting and spin coating, were explored. For such purpose, HDI-GO 4 and HDI-GO 5 samples as well as PEDOT:PSS/HDI-GO 5 nanocomposites with a HDI-GO loading of 10 wt% were tested using two solvents: DMSO and NMP. The samples with PEDOT:PSS were found to be the most opaque and the thickest ones, showing thicknesses in the range of 300–500 nm. Macroscopic segregation was observed, which likely took place during film drying and curing. A heterogeneous dispersion of graphene-based materials within a PEDOT:PSS matrix has also been previously reported in other studies, in which the nanocomposites were prepared via solution spin coating or in situ polymerization [46,62].

To assess whether the issues related to opacity and dispersion could be solved by decreasing the HDI-GO loading, drop-casting deposition was also carried out using a lower nanofiller content (1 wt%). In this case, the surface was fully covered by the nanomaterial, albeit some small agglomerates could still be observed. On the other hand, no significant influence of the solvent nature on the film morphology was found. New approaches such as surface chemical engineering could be further developed to improve the surface wetting. In addition, no significant visual differences were observed between HDI-GO 4 and HDI-GO 5 samples. Both displayed some aggregates, and did not fully cover the whole surface. Although the HDI-GO covered substrates exhibited higher transparency than HDI-GO/PEDOT:PSS nanocomposites, they were quite heterogeneous and opaque.

Representative samples prepared via spin coating technique are shown in Figure S1. In this case, the ozone surface plasma treatment was applied to improve layer deposition. Spin-coated samples are transparent, with a few small HDI-GO aggregates on the surface, showing a very different appearance from the drop-casted reference, which is clearly opaque. This suggests that spin-coating is a better method for spreading uniform thin films of graphene-based materials, in agreement with previous works dealing with solution processed GO films [63]. Furthermore, results obtained after repeating the deposition process without plasma treatment were very similar, indicating that the plasma treatment does not significantly affect HDI-GO deposition. Nonetheless, a cleaner surface is expected to improve layer deposition, since the presence of impurities can induce the nucleation of grains, hence resulting in surface heterogeneities [64].

To get better insight about the HDI-GO film formation over the substrates, samples were further investigated by UV–vis absorption spectroscopy and AFM, and the results are detailed in the following sections.

### 3.6. UV–Vis Absorption Spectroscopy

**Figure 7:**
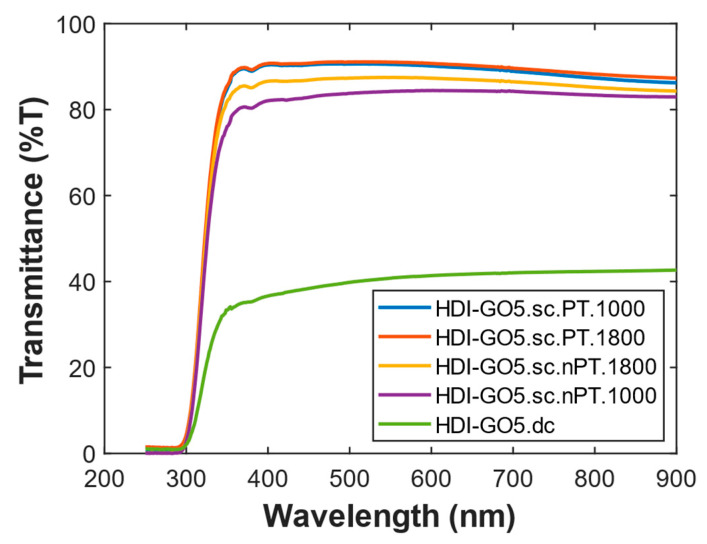
UV spectra of the samples deposited onto glass: SC—spin coated; DC—drop casted; nPT—without plasma treatment; PT—with plasma treatment.

UV–visible spectra were recorded for all the samples obtained by spin coating and for the drop-casted reference sample. The comparison of the UV–vis spectra of the samples deposited onto glass is shown in Figure 7.

As can be observed, the drop-casted sample presents around 50% less transmittance than the spin-coated ones, in agreement with the presence of a thicker and opaquer layer. Conversely, all the spin-coated samples show much higher transmittance and much closer spectra. Small differences are found between samples obtained from different deposition speeds (1000 rpm and 1800 rpm), though a higher speed led to a higher transmittance. This may be due to the formation of thinner films or lower amount of HDI-GO deposited, i.e., not covering the whole substrate. The plasma treatment also resulted in samples with higher transmittance, which may be related with thinner films or less coverage of the substrates. Similar results on film transmittance have been previously reported for CF4 plasma-treated HDI-GO-free polyethylene terephthalate (PET) thin films [65], which were related to a lower amount of defects in the film structure. However, light scattering effects caused by the presence of heterogeneities at the surface can also be at the origin of the observed small differences in the transmittance of the films.

**Figure 8:**
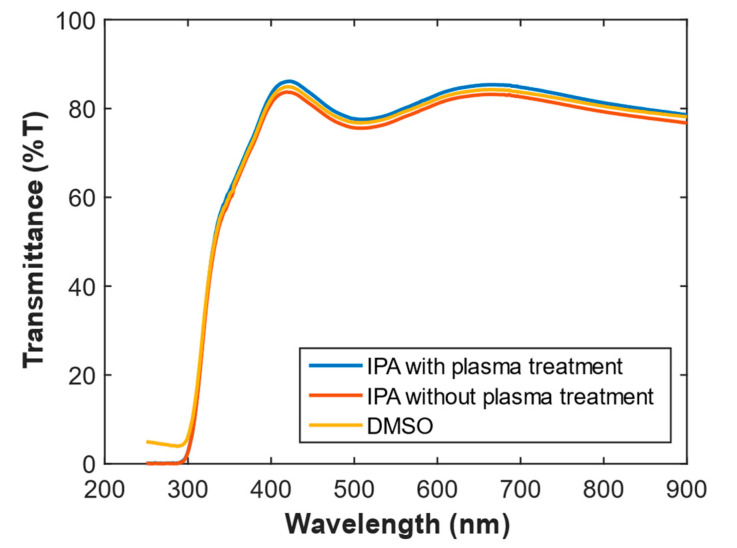
Comparison of the UV spectra of HDI-GO 5 deposited onto glass/ITO substrates using different solvents.

The effect of the solvent used for the preparation of the HDI-GO dispersions on the film transmittance was also investigated (Figure 8). DMSO or IPA were used as dispersing agents for HDI-GO 5 samples, and the resultant dispersions were deposited onto glass/ITO substrates prior to the plasma treatment; the substrate was changed in order to verify whether a different surface improved the deposition. Further, another sample was prepared using IPA though without plasma treatment (note that the use of glass/ITO implies an additional absorbance of 4–6%, hence the spectra are different from those onto glass [66]). The spectra reveal very similar transmittance values, indicating that, despite the higher polarity (not favorable to disperse the hydrophobic HDI-GO), the use of IPA results in dispersions with similar film forming properties. It would be expected that since HDI-GO is hydrophobic, the increase in the solvent polarity would not be favorable for the nanofiller dispersion, and would henceforth result in lower transmittance, due to light scattering occasioned by the small-sized aggregates.

**Figure 9:**
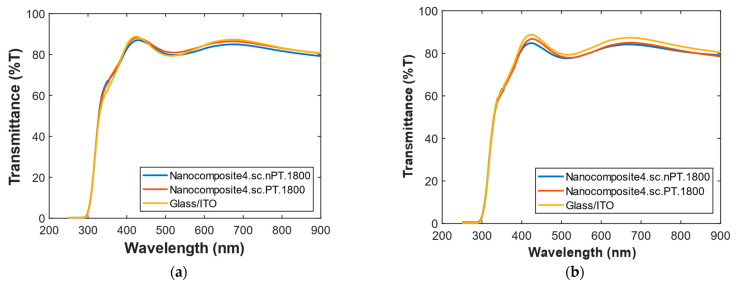
Effect of ultrasonication on the UV spectra of PEDOT:PSS/HDI-GO 4 nanocomposites deposited onto glass/ITO: (**a**) control set; (**b**) double sonication cycle. PEDOT:PSS and glass/ITO spectra are included for comparative purposes. Symbols of the legend are indicated in Figure 7.

**Figure 10:**
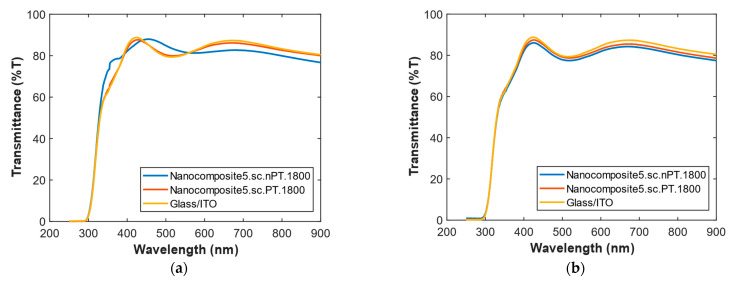
Effect of ultrasonication on the UV spectra of PEDOT:PSS/HDI-GO nanocomposites deposited onto glass/ITO: (**a**) control set; (**b**) double sonication cycle. Symbols of the legend are indicated in Figure 7.

Finally, the effect of the ultrasonication conditions for HDI-GO dispersion on the film transmittance was investigated, and the recorded UV–vis spectra for PEDOT:PSS nanocomposites with 10 wt% loading of HDI-GO 4 and HDI-GO 5 are shown in Figure 9 and Figure 10, respectively. Regarding HDI-GO 4 samples, the spectra obtained with and without plasma treatment are quite similar, though the one with plasma shows slightly higher transmittance, similarly to what was found for the samples of HDI-GO 5. The changes in transmittance found in the range of 400–650 nm are likely related to small differences in the thickness of the ITO layer or substrate [67,68].

Regarding HDI-GO 5, the spectra of samples prepared via a double cycle set (see experimental section) show scattering throughout the whole spectrum, while there is no significant scattering in the control set. Additionally, in the range of 300–570 nm, the transmittance of the sample prepared without plasma treatment was slightly higher than that of the corresponding treated sample. This is consistent with the fact that HDI-GO 5 with higher FD presents improved dispersion in polar aprotic solvents [11]: due to the increase in hydrophobicity, the presence of HDI-GO at the surface should be higher. This fact also results in poorer quality of the interface, due to the higher number of HDI-GO flakes, which is expected to result in lower transmittance.

### 3.7. AFM Results

**Figure 11:**
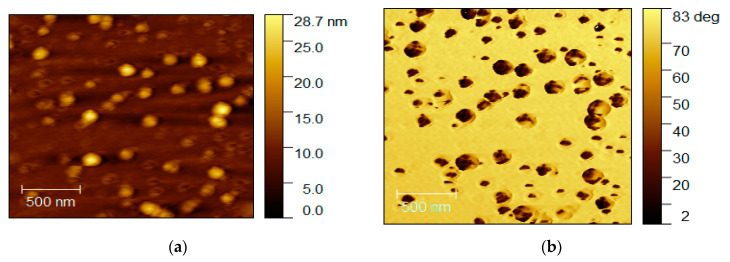
AFM images of HDI-GO 5 sample deposited over glass subjected to plasma treatment and spin-coated at 1000 rpm: (**a**) topography image; (**b**) phase image.

In order to assess the morphology of the prepared HDI-GO samples, as thin film or interfaces, AFM microscopy in the non-contact mode was used. Films resulting from the deposition of HDI-GO onto glass or glass/ITO substrates were analyzed. For the AFM study, only spin-coated samples were considered, since the drop-casted ones proved to be more heterogeneous and exhibited low transmittance. In addition, the lowest coating speed (1000 rpm) was selected, as this showed to lead to the films best covering the surface areas. The images obtained for HDI-GO 5 deposited onto glass after submitting the substrate surface to an ozone plasma treatment are displayed in Figure 11. Prior to studying the HDI-GO samples, reference images of naked glass and glass/ITO substrates were acquired, as shown in Figure S2. The glass sample (Supplementary Figure S2a,b) presents a very smooth flat surface, as expected for a clean surface of glass. In contrast, the ITO surface (Figure S2c,d) shows an irregular flake-based structure, while the phase image, not showing distinct domains, is consistent with the presence of a single material at the surface, in agreement with the results reported elsewhere [69].

From the topography and phase images acquired at different surface locations, it can be inferred that HDI-GO 5 grains are present at the glass surface. The phase image shows grains with color contrast compared to the surrounding surface, suggesting that the surface is composed of isolated grains over clean glass. It is also possible that the surrounding surface is formed by a very thin layer of HDI-GO, with different viscoelastic properties from those of the surface of the grains, and therefore resulting in a contrasting signal in the phase image. These observations corroborate that HDI-GO 5 was successfully deposited onto the glass surface as a thin layer. The grain size distribution is in the range of 100 to 250 nm, while the heights reached values close to 40 nm.

**Figure 12:**
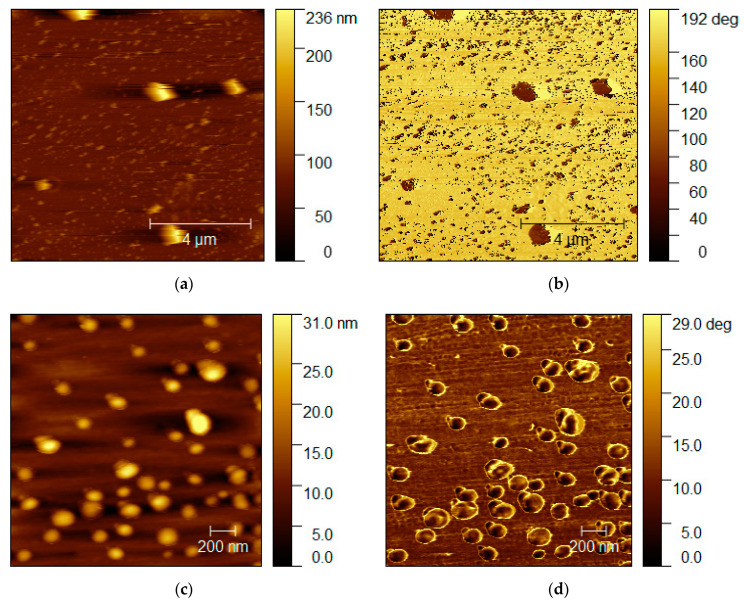
AFM images of HDI-GO5 sample spin-coated onto glass at 1000 rpm without plasma treatment, at different surface locations and different magnifications: (**a**,**c**) topography images; (**b**,**d**) phase images.

To assess the effect of surface plasma treatment on HDI-GO deposition, an HDI-GO 5 sample was also spin-coated onto an untreated glass substrate at a speed of 1000 rpm, and representative images are shown in Figure 12. Almost indistinct colors between the grains and the surrounding surface, in the phase images, can be observed in certain regions, thus suggesting that the films are composed of HDI-GO fully covering the substrate surface. The spherical shaped grains exhibit an average size close to 100 nm, which is also smaller than those found for the films deposited over substrates treated with ozone plasma, thus indicating that the non-treated glass is a more beneficial substrate for the homogeneous deposition of HDI-GO.

The effect of different solvents on the HDI-GO deposition was also investigated. Representative images of HDI-GO 5 samples deposited from DMSO solutions onto glass/ITO substrates at a coating speed of 1000 rpm and with surface plasma treatment are shown in Figure S3. In these cases, the presence of HDI-GO could not be confirmed, since the appearance of the samples was very similar to that of ITO (Figure 12a,c vs. Figure S2b). An analogous sample was prepared using IPA as solvent, and the resulting films (Figure S4) were very similar to those obtained using DMSO.

Finally, the combined effect of solvent nature and ozone plasma treatment was examined. The images of HDI-GO 5, prepared using IPA as solvent and deposited over substrates with and without plasma treatment, are compared in Figures S4 and S5. However, as inferred from UV–vis measurements, no evidence of solvent or plasma treatment effect was found, and the obtained images were quite similar in both cases. This is surprising since plasma treatment alters surface chemistry by introducing hydrophilic oxygenated groups, which should make the substrate surface more wettable and more prone to interact with aqueous solutions and solutions in polar solvents. As a result, it would be expected that the plasma treatment would promote adhesion and smooth spreading over the substrate.

### 3.8. Evaporation Essay

In order to investigate if high quality films and with full coverage of the substrates, can be obtained by thermally evaporating HDI-GO under ultra-high vacuum (ca. 10^−6^ mbar). However, HDI-GO did not evaporate, even heating up the tungsten boat in which the sample was located to a temperature that cause decomposition of most organic materials. This is consistent with the high thermal stability of HDI-GO, as confirmed by TGA analysis, and with the fact that graphene CVD production methods usually involve temperatures higher than 1000 °C. At the current state of HDI-GO materials, their potential use can be related with high thermal materials, i.e., nano-reinforcement nanomaterial for thermal composite applications [70,71,72].

## 4. Conclusions

In this study, nanocomposites made of conductive PEDOT:PSS reinforced with different amounts of HDI-functionalized GO, with two different functionalization degrees, have been manufactured, and their morphology, thermal, electrical, thermoelectrical and mechanical performance have been investigated. Outstanding improvements in electrical conductivity (more than 2-fold), thermal stability (up to 74 and 51 °C in the initial and the maximum degradation rate temperatures, respectively), Young’s modulus (up to 2.3-fold increase) and tensile strength (about 3-fold) were found. These are ascribed to the strong interaction between the modified carbon nanomaterial and the conductive polymer via hydrogen bonding, electrostatic, hydrophobic and π–π interactions. Improved thermoelectric properties were also observed for the nanocomposites, in particular for those comprising the HDI-GO with the highest FD, and an optimum combination of properties was observed at 2 wt% loading. The formation of a more densely packed and a higher degree of crystalline structure as evidenced by the sharper XRD diffractions at low diffraction angles as well as a shorter π–π stacking distance should account for the improved performance found at such loading.

HDI-GO and PEDOT:PSS/HDI-GO were deposited onto ITO and glass/ITO substrates via two different deposition techniques, drop casting and spin coating. The drop-casted samples showed a thicker and opaquer surface, while spin coating led to more homogenous films and with higher transparency. The spin-coated samples were analyzed via UV–vis spectroscopy and AFM microscopy, in order to assess the effect of the solvent nature, ultrasonication conditions, and surface treatment on the homogeneity of the films. Experimental results revealed that the modification of the preparation conditions (i.e., longer sonication time, change in the solvent polarity or the plasma treatment) hardly affects the quality of the deposited films.

Given their remarkable thermoelectric properties (Seebeck coefficient as high as 32 µV K^–1^), thermal stability (initial degradation temperature up to 207 °C) and strength (up to 120 MPa), HDI-GO-based materials show great potential to be used in thermoelectric devices and high thermal applications. This work sheds light on the understanding of material chemistry and provides a guideline to fabricate high-performance GO-based thermoelectric materials. New synthesis procedures for the nanocomposites and/or alternative surface treatments to improve the surface wetting or substrate adhesion will be investigated in the future to optimize their composition and properties (i.e., transparency) and improve their suitability for use in PSCs.

## Figures and Tables

**Table 1:** Experimental conditions used for the spin-coating of the samples.

Sample	With Plasma Treatment	No Plasma Treatment	Coating Speed (rpm)	Drying Speed (rpm)	Annealing Time (min)	Annealing Temperature (°C)
PEDOT:PSS	Deposition of sample	Clean the substrate surface with N_2_	1800	1800	10	125
GO + DMSO			1800/1000	1800/1000	30	165
GO + propan-2-ol			1800/1000	1800/1000	10–15	150

**Table 2:** Data obtained from XRD patterns and TGA thermograms of PEDOT:PSS/HDI-GO nanocomposites.

Sample	*d*(100) (Å)	*d*(010) (Å)	T_i_ (°C)	T_10_ (°C)	T_max(I,II)_ (°C)	R (wt%)
PEDOT:PSS	25.21	3.489	130	227	249, 479	6.8
PEDOT:PSS/HDI-GO 4 (0.5 wt%)	25.03	3.480	141	231	253, 465	7,1
PEDOT:PSS/HDI-GO 4 (1 wt%)	24.14	3.465	152	239	259, 487	7.9
PEDOT:PSS/HDI-GO 4 (2 wt%)	22.49	3.421	179	258	278, 533	8.4
PEDOT:PSS/HDI-GO 4 (5 wt%)	23.11	3.435	187	266	291, 550	9.7
PEDOT:PSS/HDI-GO 4 (10 wt%)	25.13	3.477	186	262	286, 547	10.3
PEDOT:PSS/HDI-GO 5 (0.5 wt%)	24.52	3.465	152	248	266, 499	7.2
PEDOT:PSS/HDI-GO 5 (1 wt%)	23.39	3.432	178	262	284, 509	7.4
PEDOT:PSS/HDI-GO 5 (2 wt%)	22.06	3.409	207	277	301, 537	8.7
PEDOT:PSS/HDI-GO 5 (5 wt%)	22.87	3.435	204	281	304, 566	9.5
PEDOT:PSS/HDI-GO 5 (10 wt%)	23.22	3.452	188	278	302, 576	10.6

*d*(100) and *d*(100): *d* spacing values of (100) and (010) planes of PEDOT:PSS. T_i_: initial degradation temperature at 2% weight loss; T_10_: temperature of 10% of weight loss. T_max_: temperature of maximum rate of weight loss. The subscripts I and II refer to the first and second degradation stages, respectively. R: residue at 700 °C.

## Data Availability

The data supporting the findings of this study are available within the article or its supplementary materials.

## Supplementary Materials

The following are available online at https://www.mdpi.com/article/10.3390/polym13091503/s1. Figure S1: Spin coating samples of HDI-GO 5 in DMSO (10 wt%); Figure S2: AFM images of the substrate reference samples; Figure S3: AFM images of HDI-GO5 spin-coated at 1000 rpm over glass/ITO treated with ozone plasma and using DMSO as the solvent, at different magnifications; Figure S4: AFM images of HDI-GO 5 spin-coated at 1000 rpm over glass/ITO treated with ozone plasma treatment and using IPA as the solvent; Figure S5: AFM images of HDI-GO 5 sample spin-coated at 1000 rpm over glass/ITO substrates without plasma treatment and using IPA as solvent.
