# Supplementary material for: Effect of HDI-Modified GO on the Thermoelectric Performance of Poly(3,4-ethylenedioxythiophene):Poly(Styrenesulfonate) Nanocomposite Films
Source: Polymers (Basel). 2021 May 7;13(9):1503. doi: 10.3390/polym13091503 (PMC8124150; doi:10.3390/polym13091503)
Supplement: Supplementary file 1 [file polymers-13-01503-s001.zip › polymers-1202000-supplementary.pdf]

Supplementary Materials

Effect of HDI-modified GO on the thermoelectric performance of poly(3,4-ethylenedioxythiophene):poly(styrenesulfonate) nanocomposite films

José A. Luceño-Sánchez [1], Ana Charas [2] and Ana M. Díez-Pascual [1,*]

[1] Universidad de Alcalá, Facultad de Ciencias, Departamento de Química Analítica, Química Física e Ingeniería Química, Ctra. Madrid-Barcelona, Km. 33.6, 28805 Alcalá de Henares, Madrid, España (Spain); jose.luceno@uah.es

[2] Instituto de Telecomunicações, Instituto Superior Técnico, Av. Rovisco Pais, P-1049-001 Lisbon, Portugal; ana.charas@lx.it.pt

* Correspondence: am.diez@uah.es; Tel.: +34-918-856-430

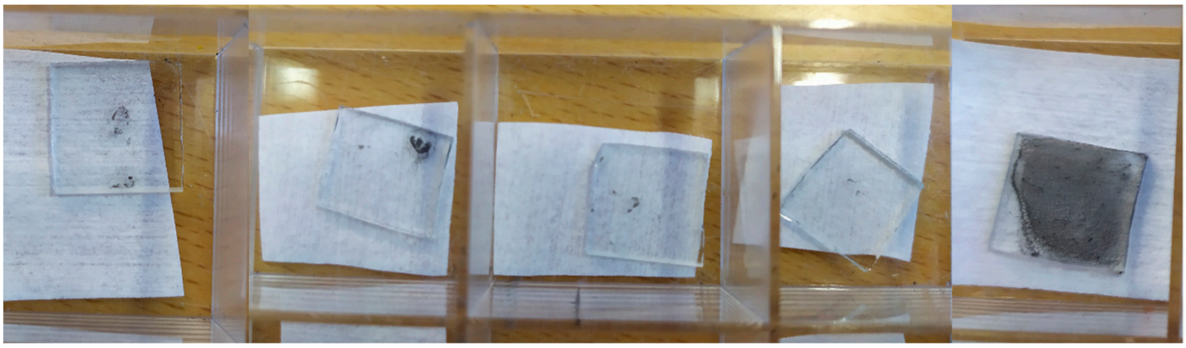

Figure S1. Spin coating samples of HDI-GO 5 in DMSO (10 wt%) (from left to right): spin-coated at 1000 rpm, substrate without ozone treatment; spin-coated at 1000 rpm, substrate with ozone treatment; spin-coated at 1800 rpm, substrate without ozone treatment; spin-coated at 1800 rpm, substrate with ozone treatment. For comparison, the drop-casted reference sample is shown in the last photo.

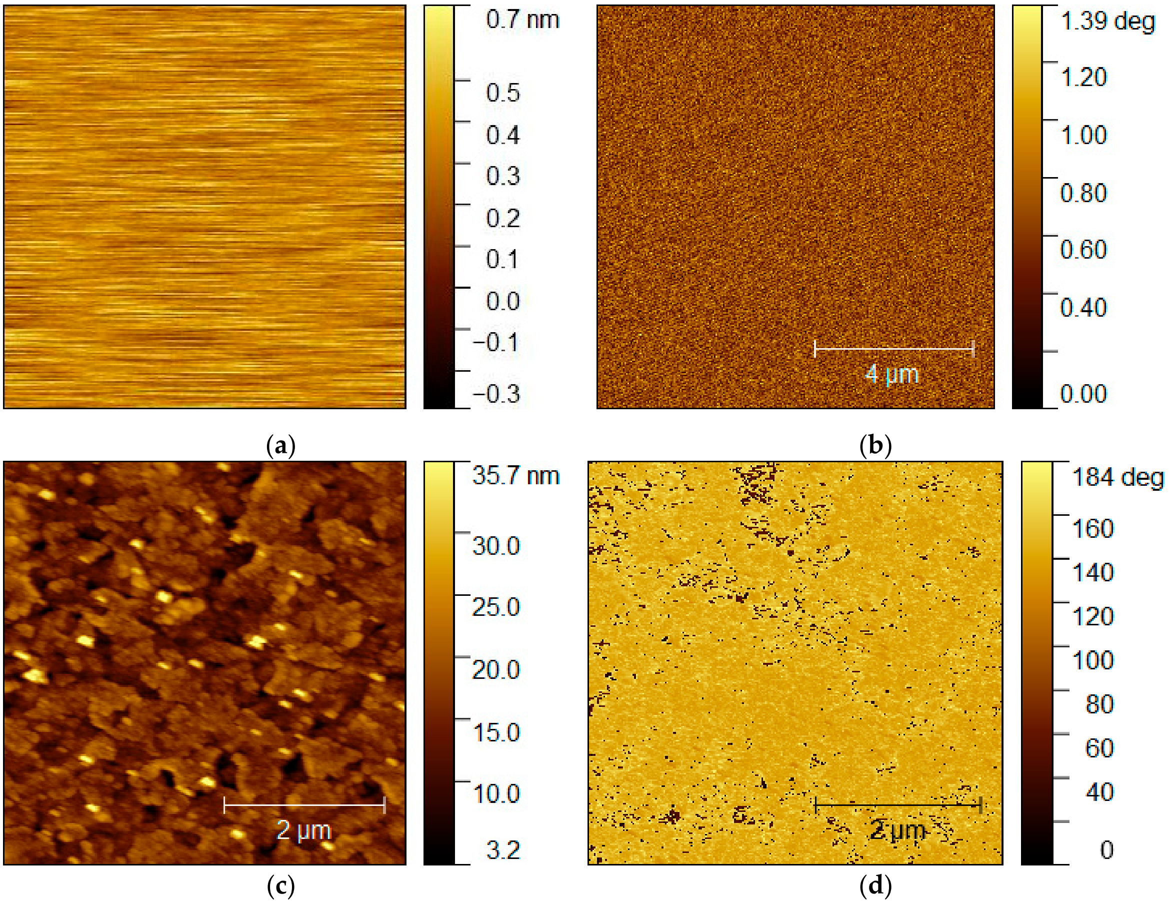

Figure S2. AFM images of the substrate reference samples: (**a**) glass 10x10 µm topography image; (**b**) glass 10x10 µm phase image; (**c**) glass/ITO 5x5 µm topography image; (**d**) glass/ITO 5x5 µm phase image.

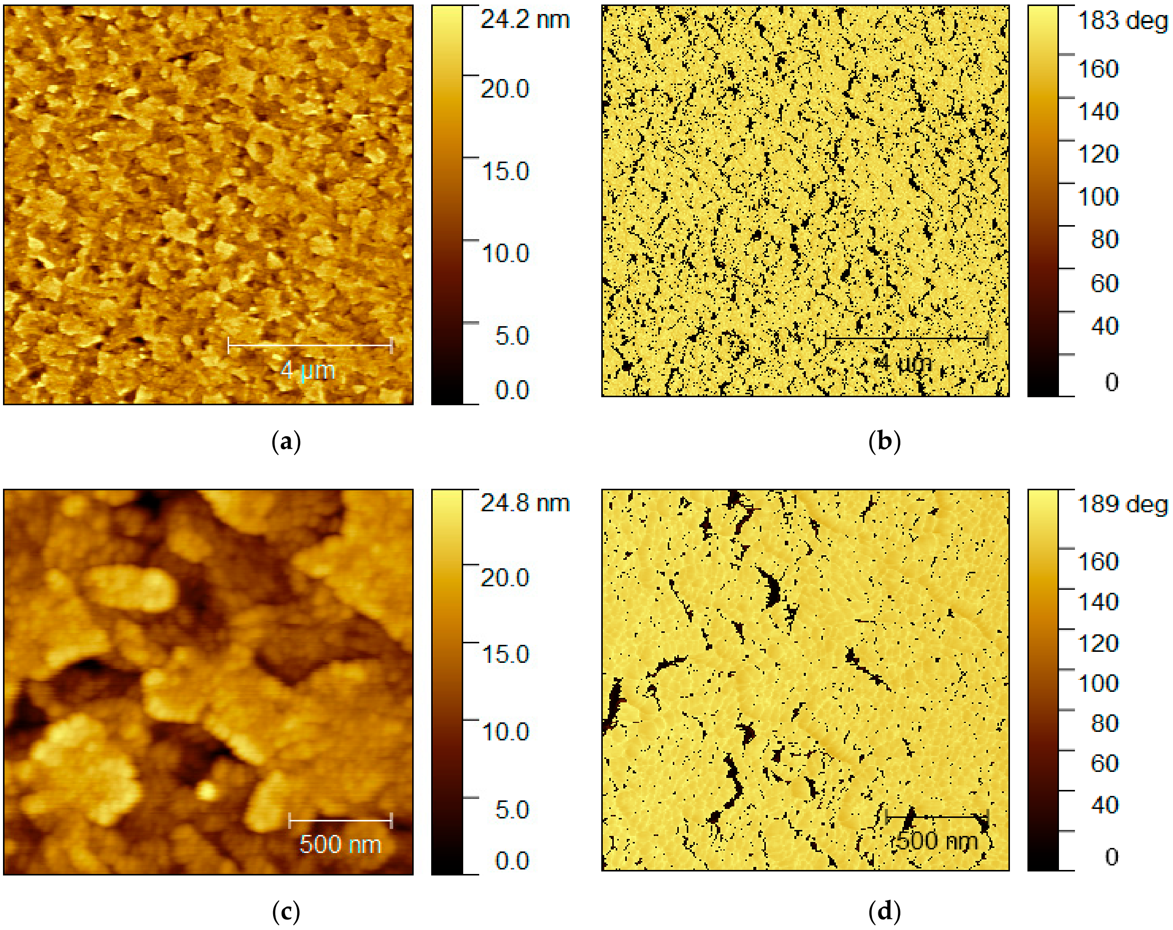

Figure S3. AFM images of HDI-GO5 spin coated at 1000 rpm over glass/ITO treated with ozone plasma and using DMSO as the solvent, at different magnifications: (**a and c**) topography images; (**b and d**) phase images.

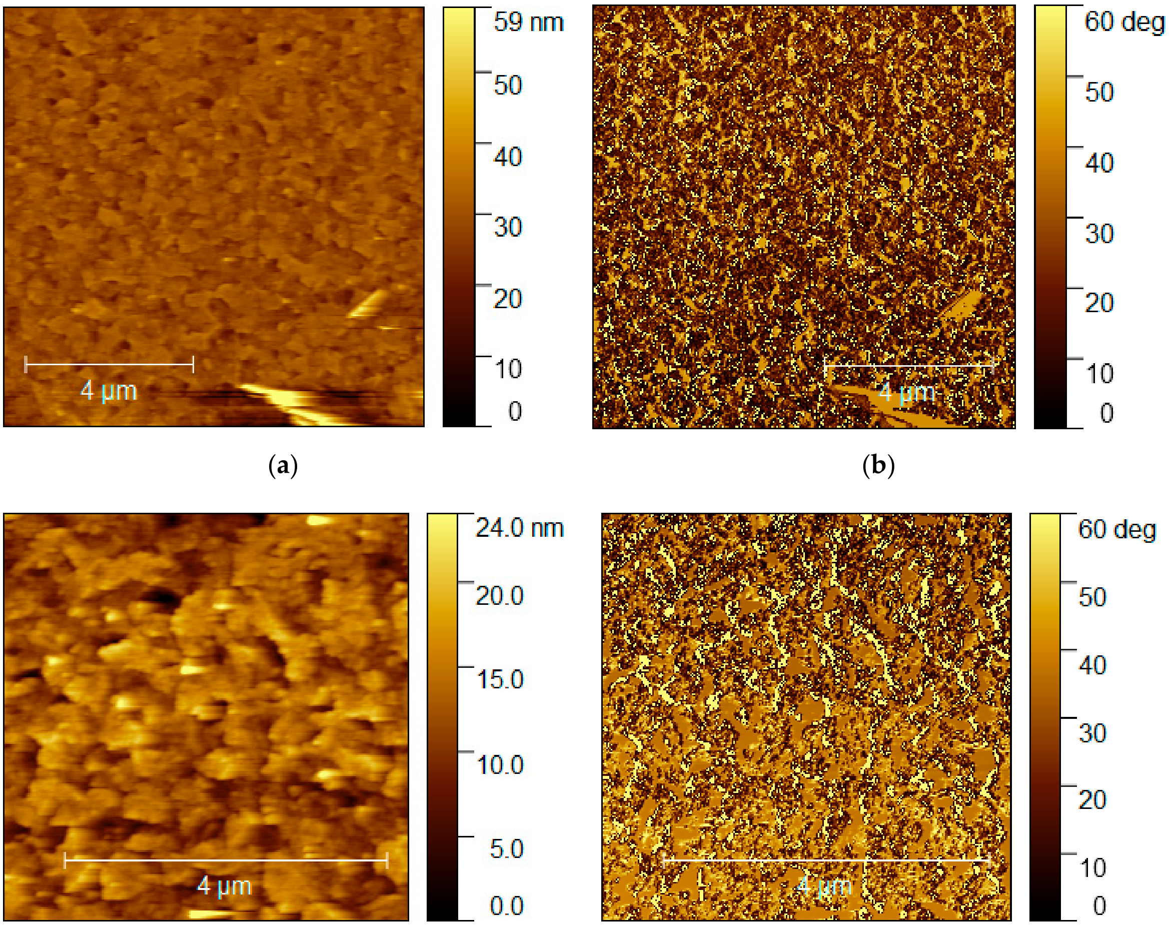

(**a**) (**b**) (**c**) (**d**)

Figure S4. AFM images of HDI-GO 5 spin coated at 1000 rpm over glass/ITO treated with ozone plasma treatment and using IPA as the solvent: (**a**) 10x10 μm topography images; (**b**) 10x10 μm phase images; (**c**) 5x5 μm topography images; (**d**) 5x5 μm phase images.

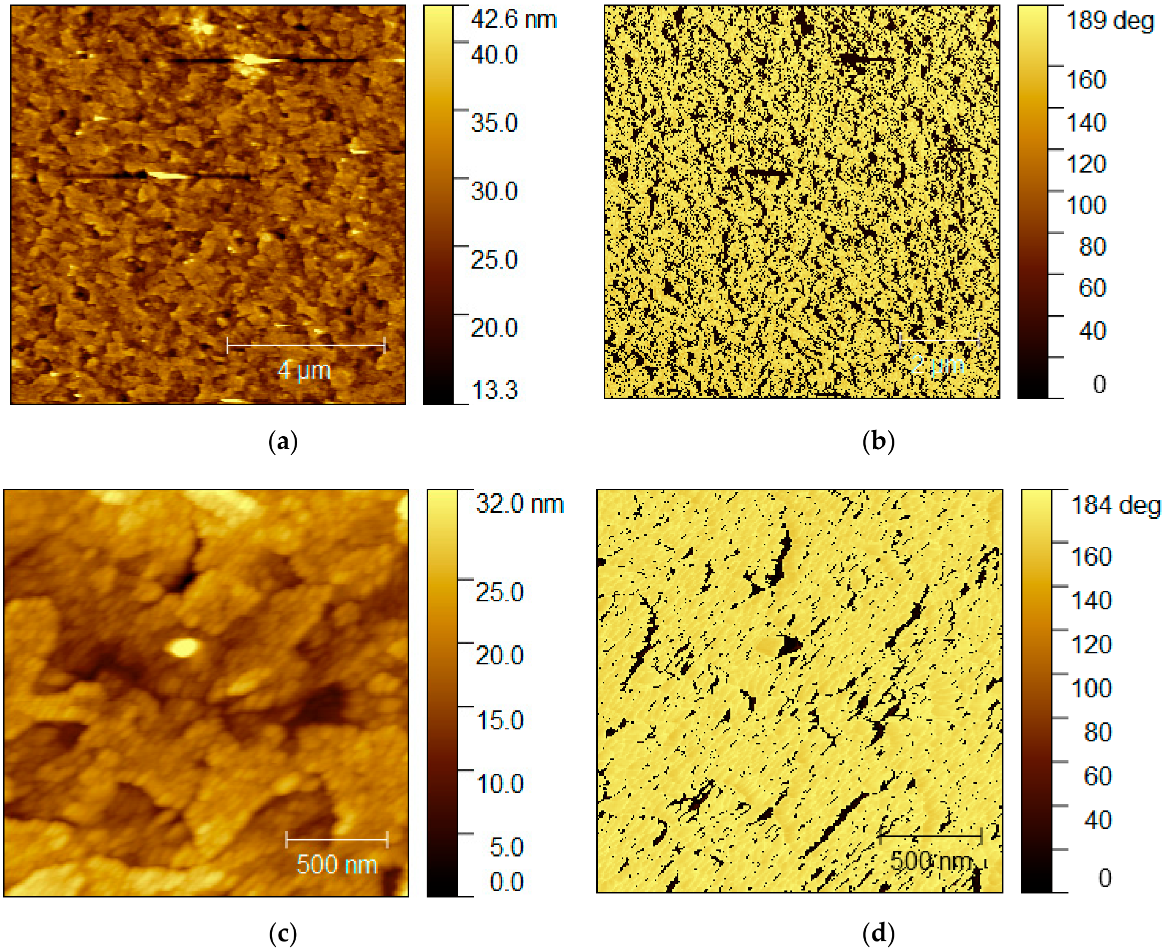

Figure S5. AFM images of HDI-GO 5 sample spin coated at 1000 rpm over glass/ITO substrates without plasma treatment and using IPA as solvent: (**a**) 10x10 µm topography micrograph; (**b**) 10x10 µm phase micrograph; (**c**) 5x5 µm topography micrograph; (**d**) 2x2 µm phase micrograph.
